# Comparative Evaluation of Sexual Behavior, Semen Characteristics and Environmental Modulation in Local Algerian and New Zealand White Rabbit Bucks

**DOI:** 10.3390/vetsci13070611

**Published:** 2026-06-25

**Authors:** Ibtissem Boulbina, Mohammed El-Amine Bekara, Hacina AinBaziz, Asma Kassoul, Cesare Castellini

**Affiliations:** 1 Laboratory of Research “Health and Animal Productions”, Higher National Veterinary School “Rabie Bouchama”, Issad Abbes Street, Oued Smar, Algiers 16059, Algeria; h.ainbaziz@ensv.dz; 2 Laboratory of Molecular Biology, Genomics and Bioinformatics, Department of Biology, Faculty of Nature and Life Sciences, University Hassiba Benbouali of Chlef, Chlef 02000, Algeria; m.bekara@univ-chlef.dz; 3 Central Biochemistry Laboratory, Mustapha University Hospital Center, Faculty of Pharmacy, University of Health Sciences, Route Nationale N36, Château Neuf, Ben Aknoun, Algiers 16028, Algeria; kassoul.asmaa@gmail.com; 4 Department of Agricultural, Food and Environmental Science, University of Perugia, Borgo XX Giugno 74, 06124 Perugia, Italy; cesare.castellini@unipg.it

**Keywords:** rabbit buck, local genetic resources, reproductive performance, semen quality, photoperiod, temperature–humidity index

## Abstract

This study aimed to characterize the reproductive performance of the local Algerian population (LAP) compared with the New Zealand White (NZW) rabbits, by evaluating sexual behavior, semen characteristics, and their modulation by environmental factors, namely photoperiod and temperature-humidity index (THI). Mature bucks (*n* = 14/breed) were monitored from January to April, with two successive ejaculates collected weekly. Sexual behavior, macroscopic and microscopic semen parameters, and testosterone concentrations were assessed. The effects of breed, ejaculate order, environmental factors, and their interactions were analyzed using Generalized Linear Mixed models. LAP and NZW bucks exhibited similar sexual behavior and blood testosterone levels (*p* > 0.05). Collection failures and ejaculate rejection causes were mainly clustered within specific individuals rather than being breed-dependent. However, LAP bucks showed higher sperm concentration (*p* = 0.01), viability (*p* = 0.02), and membrane integrity (*p* = 0.04) than NZW bucks, whereas most motility and quantitative semen traits remained comparable between breeds. Increasing photoperiod significantly improved reproductive performance (*p* < 0.05). Conversely, within the investigated range, THI mainly affected semen collection efficiency through increased urine contamination (*p* < 0.001), with limited effects on intrinsic sperm quality. Significant breed × environment interactions for sperm concentration (*p* = 0.03) suggested differential responsiveness between breeds, with LAP bucks showing a stronger positive response to increasing photoperiod and less pronounced variation under THI fluctuations. Overall, LAP bucks exhibited a more favorable seminal profile under the conditions of the present study, supporting the valorization of this local genetic resource for artificial insemination programs under Algerian conditions. Further studies are required to confirm these patterns under summer heat-stress conditions and evaluate their impact on fertility outcomes.

## Simple Summary

Artificial insemination efficiency in rabbits depends on good semen quality and the ability of males to adapt to environmental conditions. This study compared the reproductive performance of the local Algerian rabbit population with the widely used New Zealand White breed during the winter-to-spring period. Sexual behavior, semen quality, and the influence of day length and climate were evaluated. Both breeds showed similar sexual behavior and testosterone levels. However, the local breed exhibited a more favorable seminal profile. Increasing day length improved semen production and sperm quality in both breeds, whereas moderate climatic variations had limited impact on reproductive traits. The local Algerian rabbits also appeared to respond better to environmental variations than the commercial breed. These findings highlight the reproductive potential of this local genetic resource and support its use in artificial insemination programs under Algerian conditions. Further studies are needed to confirm these advantages during summer heat-stress conditions and evaluate their effects on fertility.

## 1. Introduction

The conservation and utilization of local animal genetic resources are among the strategic priorities for global livestock development, as emphasized by the Food and Agriculture Organization (FAO) through its Global Plan of Action [1,2]. Local breeds represent valuable reservoirs of genetic diversity and adaptive traits that contribute to productive efficiency and resilience under challenging environmental and management conditions [3,4]. Nevertheless, the widespread and often unregulated use of a limited number of highly selected commercial breeds has accelerated genetic dilution, placing locally adapted populations at increasing risk of extinction worldwide [5].

In Algeria, the local Algerian population (LAP) of rabbits constitutes an important component of national livestock biodiversity. This population is generally described as well adapted to local climatic conditions, low-input feeding systems, and traditional farming practices [2,6,7]. However, its genetic integrity is currently under serious threat. Recent studies indicate a progressive erosion of genetic diversity [8], likely resulting from reduced population size, limited gene flow, inbreeding, and uncontrolled crossbreeding with imported commercial breeds [5,8]. This situation highlights the urgent need for characterization and conservation efforts to safeguard this genetic resource and support its rational use in breeding programs.

While several studies have reported phenotypic, productive, and physiological traits of the local Algerian rabbit population (e.g., [9,10,11,12,13,14,15,16]), a critical knowledge gap remains regarding its male reproductive potential. Although reproductive traits have been investigated in LAP bucks, these studies were conducted either without comparison to standard commercial strains or using synthetic local lines [7,17,18,19], limiting their relevance for benchmarking reproductive performance under identical management and environmental conditions. Such information is essential for accurately assessing the reproductive value of LAP bucks, thereby supporting conservation and breeding strategies.

In rabbit reproduction, male performance is a key determinant of the efficiency, profitability, and sustainability of artificial insemination (AI) programs, which aim to produce a high number of fertile seminal doses at the lowest possible cost [20]. Beyond semen quality, male sexual behavior, such as response to sexual solicitation, ejaculation latency, and the proportion of suitable ejaculates, plays a decisive role in determining semen collection efficiency and overall reproductive and economic output. Consequently, the combined evaluation of sexual behavior and semen characteristics is essential for assessing male reproductive value and identifying genotypes best suited for AI-based breeding programs.

Environmental factors, particularly ambient temperature, relative humidity, and photoperiod, modulate male reproductive performance in rabbits. Heat stress is a well-established constraint that negatively affects libido, semen production, sperm quality and testicular morphology [21,22,23]. By contrast, reproductive performance under low-to-moderate temperature range, remains comparatively poorly documented [20]. Evaluations conducted during the winter-to-spring period, characterized by low temperature–humidity index (THI), provide an opportunity to assess the intrinsic reproductive potential of bucks under minimal thermal constraint.

Besides temperature, natural variations in photoperiod play a key role in the seasonal regulation of rabbit reproduction [24]. Changes in day length influence behavioral and reproductive traits through modulation of the hypothalamic–pituitary–gonadal axis, notably via photo-neuroendocrine pathways involving melatonin and testosterone secretion [25,26]. In European wild rabbits, reproductive activity is tightly synchronized with the seasons: testis and epididymis weights, daily sperm production, and ovarian activity vary significantly throughout the year, with peak values typically observed in March and April under Mediterranean conditions, ensuring synchronization of reproduction with favorable environmental conditions and feed availability [27]. Although domestication has attenuated the marked seasonality observed in wild populations, domestic rabbits retain a significant responsiveness to photoperiodic cues [24]. Studies conducted under controlled lighting conditions have shown that long photoperiods (14–16 h light/day) generally improve libido, sperm output and semen quality, whereas short-day regimens or prolonged darkness may impair reproductive activity [28,29,30,31]. However, the positive effect of long days can be masked by heat stress, with the THI often exerting a stronger influence on semen traits than day length alone [32,33,34]. Yet, most of these studies have focused on comparisons between seasons or artificial lighting programs, and the specific effects of natural seasonal changes in day length remain largely unexplored. Moreover, no study has simultaneously evaluated the combined effects of THI and natural photoperiod on reproductive performance of LAP bucks. Previous work has mainly focused on heat stress or broad seasonal contrasts [7,18,19], leaving a gap in our understanding of environmental influences on rabbit buck reproduction.

In this context, the present study aimed to perform a comprehensive comparative evaluation of sexual behavior, semen output, sperm quality and testosterone levels in LAP and New Zealand White (NZW) bucks during the winter-to-spring season (January-April). In addition, the relationships between these reproductive traits and key environmental factors, namely THI and natural photoperiod were analyzed. This integrative approach was designed to characterize the reproductive potential of LAP bucks in comparison with a commercial reference breed, to improve understanding of environmental modulation of male reproductive function, and to provide scientific baseline data relevant for the conservation, valorization and exploitation of this local genetic resource. It was hypothesized that LAP bucks would exhibit superior reproductive traits compared with NZW bucks due to their adaptation to local environmental conditions, and that photoperiod and THI would influence reproductive traits differently in each breed, reflecting potential breed-specific responses to environmental conditions.

## 2. Materials and Methods

### 2.1. Animals, Housing and Feeding

The study was conducted at the experimental rabbit facility of the National School of Veterinary Medicine of Algiers (Algeria). The trial spanned from January to April, covering the winter-to-spring period characterized by sub-thermoneutral to thermoneutral conditions with low thermal constraints for rabbits. The housing facility covered an area of 72 m^2^ and was of solid construction with a metal-frame roof structure. Natural ventilation was provided by a continuous ridge vent along the length of the building roof and ten windows. Lighting was exclusively natural and no heating or cooling systems were used. The experiment involved clinically healthy bucks aged 12–15 months, including 14 Local Algerian Population (LAP; mean body weight = 3.2 ± 0.48 kg) and 14 New Zealand White (NZW; mean body weight = 3.6 ± 0.18 kg) males. Sample size was determined based on animal availability and was consistent with previous rabbit reproductive studies [32,33,34]. All animals were housed individually in flat-deck cages to facilitate semen collection, fed commercial pellets ad libitum (dry matter 97.2%, crude protein 14%, crude fat 2.8%, crude fiber 13.5%, and minerals 8.1%), and had free access to drinking water.

### 2.2. Semen Collection and Assessment of Ejaculatory Response

All rabbit bucks underwent a two-week training period for semen collection [35]. After that, semen was collected once a week in the morning using a lubricated and pre-warmed artificial vagina (Importvet, Barcelona, Spain; REF 10.803). During each collection session, two successive ejaculates were obtained from each buck, with an interval of 20–30 min [36]. Two teaser does of the same breed were used and rotated across sessions. Bucks were allowed a total of 5 min to ejaculate using up to two different teasers; failure to ejaculate within this period was recorded as a failed collection.

Libido was quantified using latency measures recorded with a stopwatch (seconds): T1 (mounting latency) was defined as the time interval between the introduction of the teaser doe into the male’s cage and the first mounting attempt, and T2 (ejaculation latency) as the time interval between the first mount and ejaculation. The total latency (T total) was calculated as the time elapsed from the introduction of the teaser doe to ejaculation.

Total number of solicitations (TNS) and number of collections (NC) were recorded and the response to solicitation was calculated as RS = (NC/TNS) × 100.

### 2.3. Macroscopic Traits and Classification of Ejaculates

Immediately after collection, ejaculates were classified as either useful (UE) or discarded. Ejaculates were discarded if they exhibited urine contamination (UR), presence of blood (BL), or a volume ≤0.20 mL (VL). The presence or absence of gel plugs was also recorded (0 or 1).

The following ratios were calculated from the previously mentioned traits:

– Useful collection rate, UC = (UE/NC) × 100.
– Urine rate, UR = (UR/NC) × 100.
– Blood rate, BL = (BL/NC) × 100.
– Volume ≤ 0.20 mL rate, VL = (VL/NC) × 100.
– Gel plugs rate, G = (G/NC) × 100.

For each useful ejaculate, the following macroscopic traits were recorded: total ejaculate volume (mL) and gel-free volume (mL) using a graduated conical tube (IMV Technologies, L’Aigle, France; REF 005349), and pH, which was measured using a digital pH meter (HI98100; Hanna Instruments, Woonsocket, RI, USA).

### 2.4. Laboratory Semen Analysis

Due to volume limitations, the two successive ejaculates collected from each buck within the same week were pooled after macroscopic evaluation. All microscopic sperm analyses were subsequently performed on this pooled sample to obtain a single, representative measure of seminal quality per buck.

#### 2.4.1. Sperm Motility

Sperm motility was assessed by visual estimation using a light microscope (Olympus CX21FS2; Olympus Corporation, Tokyo, Japan) equipped with a heated stage maintained at 37 °C, according to previously described methods [37,38]. Mass motility (MM) was evaluated on a drop of raw semen at ×100 magnification and scored on a 0 (no sperm movement) to 9 (vigorous waves with a pronounced whirlwind appearance) scale. Subsequently, a drop of diluted semen from the same sample was examined at ×400 magnification to assess the percentage of total motile spermatozoa (% Motility), as well as individual motility (IM), scored from 0 (immotile spermatozoa) to 4 (fast, progressive, linear movement). Five microscopic fields were evaluated for each sample, and the mean of these observations was recorded as the final motility value.

#### 2.4.2. Sperm Concentration and Derived Indices

Sperm concentration was determined using a Thoma hemocytometer (Marienfeld Superior GmbH & Co.KG, Lauda-Königshofen, Germany; REF 0640710) after 1:200 dilution of semen in formol–physiological saline solution [36]. Spermatozoa were counted microscopically at ×400 magnification. The total sperm number (TSN) was calculated as the product of sperm concentration and pooled ejaculate volume. The total motile spermatozoa (TMS) was calculated as TSN multiplied by the percentage of motile spermatozoa. The number of artificial insemination doses per session was estimated as the ratio of TMS divided by 10 million spermatozoa per dose [39].

#### 2.4.3. Plasma Membrane Integrity

Plasma membrane integrity was evaluated using the hypo-osmotic swelling test (HOST). Briefly, 100 µL of semen were incubated with 900 µL of 60 mOsm/kg fructose solution at 37 °C for 30 min [40]. After incubation, a drop of the mixture was examined microscopically at ×400 magnification. A total of 200 spermatozoa were evaluated per sample and classified as swollen (intact membrane) or non-swollen (damaged membrane). Results were expressed as the percentage of spermatozoa with intact plasma membranes (InMb).

#### 2.4.4. Viability and Sperm Abnormalities

Sperm viability and total sperm abnormalities were assessed using eosin–nigrosin staining (RAL Diagnostics, Martillac, France) [40]. 10 µL of semen and 20 µL of eosine–nigrosin stain, smeared on a clean glass slide, and evaluated under a microscope at ×1000 magnification using oil immersion. Viable spermatozoa remained unstained, while non-viable cells exhibited pink-stained cytoplasm. A total of 200 spermatozoa per slide were counted to determine the percentages of live spermatozoa and abnormal spermatozoa [41]. Morphological abnormalities were recorded without distinction of specific defect categories.

### 2.5. Blood Sampling and Testosterone Assay

Testosterone concentrations were determined in a subset of 7 bucks per breed, consistent with sample sizes commonly used in rabbit endocrine studies [34,42]. The same individuals were sampled during the first week (January) and the last week (April) of the experimental period. Blood samples were collected from the marginal ear vein using sterile dry tubes between 09:00 and 10:00 a.m. Samples were allowed to clot at room temperature and then centrifuged at 5000 rpm for 10 min. Serum was harvested and stored at −20 °C until analysis. Serum testosterone concentrations were measured by ECLIA (ElectroChemiLuminescence ImmunoAssay) using Elecsys Testosterone II kit (Roche Diagnostics GmbH, Mannheim, Germany; REF 08946353190) on a Cobas e 411 automated analyzer (Roche Diagnostics, Mannheim, Germany). This second-generation assay is based on a competitive binding principle using a monoclonal antibody specifically directed against testosterone. The analysis was performed directly on raw serum following the manufacturer’s instructions. The lower limit of detection was 0.025 ng/mL.

### 2.6. Environmental Data Records

During the experimental period, ambient temperature and relative humidity in the rabbitry were recorded twice daily, in the morning (08:00) and during the afternoon (14:00–15:00), using a digital thermo-hygrometer (HI9565; Hanna Instruments, Woonsocket, RI, USA). The mean daily temperature and relative humidity were then used to calculate the temperature–humidity index (THI) according to Marai et al. [43]:

THI = db°C − [(0.31 − 0.31 × RH) (db°C − 14.4)],

where db°C is the dry-bulb temperature (°C) and RH is relative humidity.

Rabbit bucks were kept under natural photoperiod conditions, and day length (hours of light/day) was calculated using the “getSunlightTimes” function from the R package “suncalc” (version 0.5.1), based on the geographic coordinates of the experimental location and the corresponding experimental dates. Day length was defined as the interval between sunrise and sunset. Monthly average of temperature, relative humidity, THI, and photoperiod during the experimental period are shown in Table 1.

### 2.7. Statistical Analysis

Quantitative variables were expressed as mean ± standard deviation (SD), and qualitative variables as percentages. The effects of breed, ejaculate order, photoperiod, and Temperature–Humidity Index (THI) on sexual behavior, semen production, and sperm quality traits were analyzed using generalized linear mixed models (GLMM). In these models, breed, ejaculate order, photoperiod, and THI were included as fixed effects. Repeated measurements, nested within buck, were included as a random effect to account for within-animal variability. Statistically significant interactions were retained in the final models.

Continuous outcomes were analyzed assuming a Gaussian distribution with an identity link function, whereas binary outcomes (response to solicitation, useful ejaculates, urine contamination, blood contamination, ejaculate volume ≤ 0.2 mL, and presence of gel plug) were modeled using a binomial distribution with a logit link function.

For continuous response variables, the effects of photoperiod and THI are reported as regression coefficients (β) with their standard errors (SE). These coefficients represent the expected mean change in the response variable associated with a one-hour increase in photoperiod or a one-unit increase in THI. For binary response variables, effects are reported as odds ratios (OR), obtained by exponentiating the model estimates. The OR represents the multiplicative change in the odds of the event occurring per one-hour increase in photoperiod or one-unit increase in THI. An OR > 1 indicates increased odds of occurrence, whereas an OR < 1 indicates decreased odds of occurrence.

Based on preliminary evaluation, weekly mean THI (7-day mean preceding collection) was used for semen characteristics, while daily THI (collection day) was used for sexual behavior and ejaculate traits. Weekly photoperiod (7-day mean) was included to account for cumulative photic stimulation on the hypothalamic–pituitary–gonadal axis.

Testosterone data were analyzed using a GLMM with animal as a random effect to account for repeated measures. Fixed effects included breed, sampling week (January vs. April), and their interaction.

Model assumptions for quantitative outcomes were verified using visual inspection of residuals for normality and homoscedasticity. Data transformations were applied when necessary. Statistical significance was set at *p* < 0.05, and all analyses were performed using R software (version 4.1.1).

## 3. Results

### 3.1. Sexual Behavior and Semen Collection Efficiency

Sexual behavior and semen collection efficiency were influenced by breed, ejaculate order, photoperiod, and, to a lesser extent, THI (Table 2 and Table 3).

Out of 814 solicitations, both breeds exhibited high sexual performance, with >95% RS, >90% UC and good libido (T1, T2, and T total), showing no significant inter-breed differences. However, LAP bucks were significantly more prone to urine contamination (3.5% vs. 1.5% in NZW).

The first ejaculate was characterized by a higher RS (99.0% vs. 95.8%) and longer sexual response times (T1, T2 and T total) compared to the second. UR was also higher in the first collection (3.23% vs. 1.79%, *p* < 0.05), whereas BL and VL were similar between ejaculates.

Photoperiod exerted a marked effect on sexual behavior. Increasing day length significantly shortened all reaction times (*p* < 0.001), indicating a faster sexual response. Furthermore, longer photoperiods were associated with improved UC rate (OR = 2.68, *p* < 0.001) and reduced UR (OR = 0.47, *p* < 0.001). In contrast, THI primarily affected collection quality rather than libido, being negatively associated with UC rate (OR = 0.80, *p* < 0.05) and positively with UR (OR = 1.14, *p* < 0.001), whereas no significant effects were detected for RS, BL or VL.

### 3.2. Ejaculate Macroscopic Traits

Gel plug presence, ejaculate volume traits and pH were affected by breed, ejaculate order and photoperiod, with limited influence of THI (Table 2 and Table 3).

LAP bucks exhibited a significantly higher secretory activity compared to the NZW group, characterized by a larger total ejaculate volume (1.29 ± 0.86 vs. 0.96 ± 0.47 mL; *p* < 0.05). This difference was primarily attributed to a three-fold increase in gel plug incidence (38.1% vs. 12.4%) and a significantly higher gel volume in the local breed (1.25 ± 1.49 mL vs. 0.58 ± 0.30 mL). Conversely, breed had no significant impact on gel-free volume or semen pH.

Ejaculate order influenced volume distribution, with the first ejaculate being larger (1.24 mL vs. 1.01 mL) and more frequently containing gel plugs (34.8% vs. 15.2%).

Regarding environmental cues, increasing photoperiod was positively associated with gel plug occurrence (OR = 1.42, *p* < 0.05) and a significant reduction in semen pH (*β* = −0.13, *p* < 0.001). Conversely, THI had a negligible impact on volume traits but showed a slight positive correlation with pH (*β* = 0.01, *p* < 0.05).

### 3.3. Sperm Quality and Semen Output (Pooled Samples)

Sperm microscopic traits and semen output were influenced by breed and photoperiod, whereas THI showed only limited effects on the evaluated quantitative traits (Table 4).

Genetic background significantly impacted several sperm traits. LAP bucks exhibited higher sperm concentration compared to NZW bucks (*p* = 0.01). In addition, sperm viability (*p* = 0.02) and membrane integrity (*p* = 0.04) were significantly higher in LAP bucks. A tendency towards higher sperm % motility (*p* = 0.07) and a higher number of AI doses per collection session (*p* = 0.08) was also observed in LAP bucks, although these differences did not reach statistical significance.

Photoperiod showed the most consistent associations with sperm quality and semen output. Increasing day length improved sperm motility parameters (*p* < 0.05), viability, and membrane integrity (*p* < 0.001), and increased ejaculate volume per collection session (*p* < 0.001). It also positively affected sperm concentration (*p* < 0.01), TSN, TMS, and the number of AI doses (*p* < 0.001). However, the percentage of abnormal spermatozoa was not significantly affected.

The THI had no significant effect on qualitative sperm traits. However, it exerted a significant negative effect on semen output parameters. Increases in THI were associated with decreases in TSN (*β* = −39.82 ± 17.56, *p* = 0.02), TMS (*β* = −36.35 ± 15.49, *p* = 0.02), number of AI doses (*β* = −3.63 ± 1.55, *p* = 0.02), and pooled volume per collection session (*β* = −0.06 ± 0.02, *p* = 0.01).

Notably, significant breed × photoperiod and breed × THI interactions were observed exclusively for sperm concentration (Table 5). The positive effect of photoperiod was stronger in LAP than in NZW bucks (*β* = +61.39 vs. β = +18.66; *p* = 0.03). Conversely, THI showed a more pronounced negative effect in NZW than in LAP bucks (*β* = −26.71 vs. *β* = −9.23; *p* = 0.03).

### 3.4. Testosterone Profile

**Figure 1:**
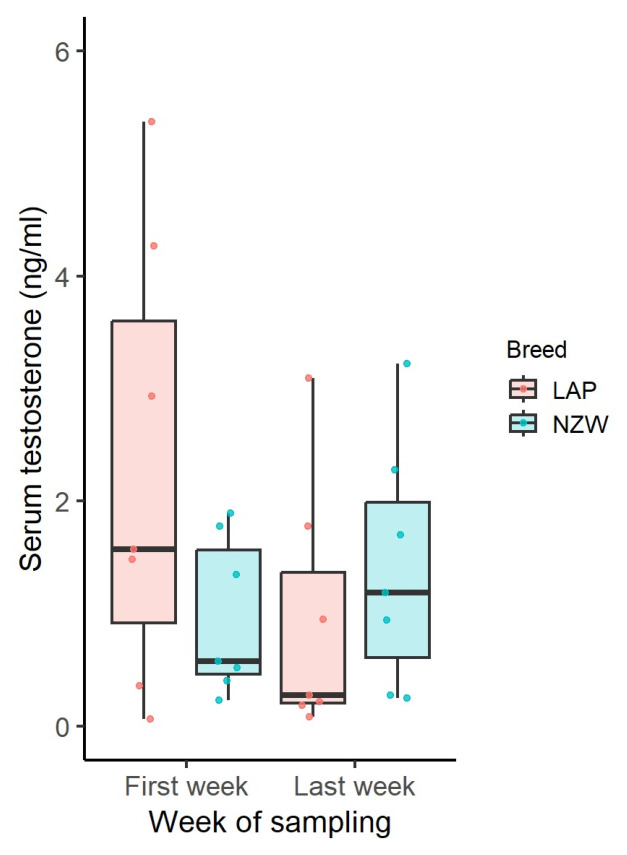
Serum testosterone concentrations in LAP and NZW buck rabbits during the first week (January) and at the last week (April) of the study. Boxplots represent the median, interquartile range (box), and range (whiskers). Individual data points are shown as jittered dots to visualize distribution and variability. No significant effects of breed, sampling week, or their interaction were detected (*p* > 0.05).

Serum testosterone concentrations (Figure 1) were not significantly affected by breed, sampling time (week), or their interaction (*p* > 0.05). Mean testosterone levels were comparable between LAP and NZW bucks (1.62 ± 1.70 vs. 1.19 ± 0.90 ng/mL), and no significant differences were observed between the beginning and the end of the experimental period (1.63 ± 1.59 vs. 1.17 ± 1.09 ng/mL).

## 4. Discussion

As far as we are aware, the present study provides the first comprehensive comparative evaluation of sexual behavior, semen output, and sperm quality in local Algerian rabbit (LAP) bucks in comparison with a commercial breed (New Zealand White), while simultaneously assessing the effects of photoperiod and THI under winter-to-spring conditions. The results indicate that LAP bucks display reproductive traits comparable to, and in some aspects more favorable than, NZW bucks under the conditions of the present study. Photoperiod was the environmental factor most consistently associated with variation in reproductive traits, whereas THI showed only limited effects within the recorded temperature range.

### 4.1. Sexual Behavior, Collection Efficiency and Ejaculate Order

Male sexual behavior is a key determinant of productivity in semen collection centers, as it directly influences collection efficiency and the proportion of functional ejaculates. In the present study, both LAP and NZW bucks exhibited high collection and useful ejaculate rates (>95% and >90%, respectively). These values fall within the upper range reported in the literature, where collection rates range from 87 to 95%, while useful ejaculate rates show greater variability depending on genotype and rejection criteria, ranging from approximately 50% to over 90% [44,45,46,47,48]. The lack of a significant breed effect indicates that LAP bucks generally respond well to routine semen collection.

However, detailed observation revealed marked inter-individual variability despite this high overall performance. Collection refusals were not a breed trait but were clustered within a few individuals, particularly among the LAP group. These events primarily involved complete mounting without ejaculation, suggesting ejaculatory failure rather than reduced libido, a finding supported by similar latency parameters between breeds. This pattern likely reflects incomplete adaptation to the artificial vagina in a small subset of bucks, as reported for other strains during early collection periods [48]. A similar pattern was observed regarding ejaculate rejection. Low volume was the primary reason for elimination in both breeds, driven by specific individuals. While urine contamination was more frequent in LAP bucks, it remained concentrated within a few males, accounting for the majority of contaminated samples. This is consistent with earlier studies [17,48], and may reflect individual susceptibility related to impaired neurophysiological coordination [48] or stress responsiveness during collection. In contrast, blood contamination remained rare in both breeds, consistent with previous reports [45], and likely represents a minor technical issue rather than a biological limitation to semen collection efficiency.

Overall, these findings highlight that individual variability, rather than breed per se, is likely the major determinant of semen collection efficiency. This emphasizes the importance of adequate individual-based management and training, particularly when integrating local breeds into structured AI programs.

Regarding the collection process, ejaculate order also influenced both behavioral and ejaculatory traits. The first ejaculate was characterized by a higher collection rate and greater total volume, mainly due to increased gel plug formation, likely reflecting maximal accessory gland reserves. In contrast, the second ejaculate benefited from residual neuroendocrine stimulation, resulting in shorter reaction times but reduced gel secretion, consistent with partial depletion of accessory gland contents. Urine contamination was more frequent in the first ejaculate.

Although ejaculate-specific differences in sperm quality have been reported in previous studies [46,48,49,50], microscopic parameters were not analyzed separately in the present study because ejaculates were pooled for processing. Generally, our findings are consistent with those of García-Tomás et al. [46] and support the routine collection of two successive ejaculates to optimize total semen output in rabbit AI programs.

### 4.2. Breed-Related Differences

While behavioral parameters and collection efficiency were comparable between breeds, significant divergences appeared when assessing microscopic sperm traits. LAP bucks exhibited higher sperm viability and membrane integrity (HOS test), together with higher sperm concentration compared with NZW bucks, whereas most quantitative semen traits remained similar between breeds. The higher viability and membrane integrity observed in LAP spermatozoa indicate enhanced structural stability and a greater capacity to withstand physiological challenges, which is critical given that the plasma membrane is a primary target of damage during semen handling and storage [51]. This qualitative advantage may, reflect a specific membrane lipid composition or more efficient antioxidant defense system in LAP sperm. In support of the latter hypothesis, our previous work using a subset of the same bucks showed higher catalase activity in LAP seminal plasma compared with NZW [52], suggesting a more efficient antioxidant defense that may contribute to membrane preservation against premature degradation. Direct measurements of membrane lipid profiles and additional antioxidant markers would be required to confirm these hypotheses.

These differences are likely related to genetic background and selection history. Commercial lines such as NZW have undergone intensive selection for growth and production traits, which may indirectly affect reproductive characteristics, whereas local breeds such as LAP may have retained more balanced reproductive functions. Previous studies have reported negative relationships between body weight and semen quality traits in rabbits, indicating that heavier bucks may exhibit reduced reproductive efficiency [53], while selection for production traits has been associated with divergent physiological responses in reproductive parameters [45].

Interestingly, a breed-related phenotypic difference emerged in the secretory activity of accessory sex glands. LAP bucks produced ejaculates with higher frequency of gel plugs than NZW bucks (38.1% vs. 12.4%), and the volume of the gel fraction was also markedly greater in LAP males (1.25 mL vs. 0.58 mL). This finding is consistent with previous observations in LAP rabbits [17,18], and exceeds values reported in other rabbit genotypes [20,46,54], suggesting that this trait may represent a stable phenotypic characteristic of this population rather than a sporadic event. A previous study has documented variations in gel plug formation among different rabbit lines, likely reflecting genetic differences in seminal vesicle secretory activity [46]. From a physiological perspective, the gel fraction is a normal and androgen-dependent component of rabbit semen. It has been suggested to contribute to sperm retention within the female reproductive tract and to modulate post-copulatory processes, although its precise functional role in rabbits remains incompletely understood [55]. Under AI conditions, Theau-Clément et al. [56] reported that although ejaculates containing gel plugs are excluded by some AI centers, this characteristic does not negatively affect fertility or prolificacy outcomes. Their removal, however, is a time-consuming step in semen preparation. Consequently, the higher frequency of gel plugs in LAP compared to NZW bucks may increase sample processing time and workload in AI centers. Despite this higher prevalence, the gel-free semen volume did not differ between breeds.

Importantly, even though these differences in both sperm functionality and accessory gland output, no significant variations were observed between breeds regarding circulating testosterone levels or sexual behavior parameters. This suggests that the observed reproductive differences are unlikely to be explained by systemic androgen levels alone, aligning with previous reports that show no clear relationship between plasma testosterone and spermatogenic efficiency in rabbits once basal levels are reached [57]. Instead, breed-specific differences in local regulatory mechanisms, target tissue sensitivity (particularly within the accessory sex glands) and the biochemical environment of seminal plasma likely play a more decisive role in shaping semen characteristics. However, these possibilities remain to be confirmed by testing.

Taken together, these findings indicate that reproductive performance in both breeds remained within the normal physiological range [21,58]. Nevertheless, LAP bucks exhibited a more favorable qualitative seminal profile, which may have practical implications for semen preservation and reproductive biotechnologies, although further studies are required to confirm its direct impact on fertility outcomes.

### 4.3. Environmental Influence

#### 4.3.1. Photoperiod

Among the environmental variables evaluated, photoperiod showed the most consistent associations with reproductive performance in the present study. The progressive increase in natural day length from winter to spring was associated with marked improvements in both sexual behavior and semen production and quality. These findings highlight an important role of photoperiod in modulating male reproductive function under cold to moderate environmental conditions in the Mediterranean–North African region.

These results are consistent with earlier studies demonstrating seasonal variation in the reproductive activity of wild European rabbits, where maximal testicular development and spermatogenic activity occur during spring under increasing day length [59,60,61]. Controlled experiments have similarly shown that exposure to long photoperiods (14 or 16 h light/day) enhances libido, sperm production, and semen quality compared with short-day conditions [28,29,30]. Conversely, extreme light deprivation has been associated with testicular alterations, increased sperm abnormalities, and reduced gonadotropin levels [31].

In contrast, Roca et al. [32] found no clear effect of seasonal photoperiod variation on semen traits in Spain, nor any advantage of artificial long-day exposure over natural conditions. However, their study included summer periods with elevated THI, where heat stress influenced semen quality more than photoperiod. This suggests that thermal stress can mask the stimulatory effect of increasing day length. Supporting this, Fouda et al. [34] showed that under severe summer heat stress in Egypt, shorter day lengths combined with melatonin supplementation improved semen quality, highlighting melatonin’s antioxidant and cytoprotective roles when oxidative stress dominates. Collectively, these findings do not contradict our results but demonstrate that the direction and magnitude of photoperiodic effects are highly dependent on the thermal context.

Interestingly, testosterone concentrations did not change significantly despite improved reproductive function, suggesting photoperiodic effects are not solely mediated by androgen increases. One hypothesis is that enhanced testicular sensitivity or improved Sertoli cell function may be involved [62,63].

Additionally, increased pooled, but not individual, ejaculate volume suggests improved collection efficiency rather than increased secretion due to lower urine contamination.

#### 4.3.2. THI Under Low-to-Moderate Temperature Conditions

In contrast to photoperiod, THI had no significant effect on key reproductive traits. Sperm concentration, ejaculate volume, sperm motility, viability, membrane integrity, morphological abnormalities, as well as sexual behavior parameters, remained unaffected across the range of THI values studied (10–22).

However, THI significantly affected semen output parameters, including total sperm number, total motile sperm count, and the number of AI doses. This effect was primarily driven by a decrease in pooled volume per collection, while ejaculate volume itself remained unchanged. This pattern suggests that THI did not affect sperm production directly but rather reduced effective semen yield through an increased proportion of ejaculates rejected due to urine contamination. Similar observations have been reported previously, where moderate increases in THI were associated with higher rates of ejaculate contamination [20]. As this phenomenon tends to be strongly clustered within a subset of susceptible bucks, the observed THI effect likely reflects increased sensitivity in predisposed individuals rather than a generalized thermal impact on reproductive function.

These findings indicate that, under low-to-moderate temperature conditions, neither spermatogenesis, epididymal reserves, nor accessory gland secretory activity were impaired. This interpretation is supported by previous studies conducted under similar environmental conditions. García-Tomás et al. [20], reported stable sperm concentration across THI values ranging from 14 to 23, while Roca et al. [32] and Nizza et al. [49] observed minimal effects of moderate temperatures on semen characteristics, with significant declines occurring only under heat-stress conditions.

Although photoperiod and THI were positively correlated during the study period (*r* = 0.83; *p* < 0.001), reflecting their concurrent increase from winter to spring, THI remained below heat-stress thresholds in rabbits [43], and showed limited associations with reproductive traits in the present study. In contrast, photoperiod was associated with a broader range of reproductive responses, suggesting that it was the environmental factor most consistently associated with the reproductive changes observed during the experimental period.

#### 4.3.3. Breed × Environment Interactions

Interestingly, significant breed × environment interactions for sperm concentration indicate distinct response between LAP and NZW bucks, aligning with well-documented genotype × environment effects on reproductive traits [64]. The photoperiod interaction was particularly evident; LAP bucks showed a greater response to increasing day length, suggesting breed-specific differences in sensitivity to photoperiodic cues. This aligns with reported variations in photoperiodic responsiveness among rabbit populations [27,65]. However, this effect was not uniform across all semen traits; its biological impact appears to be trait-dependent.

Although THI did not exert a significant overall effect, its interaction with breed suggests that even moderate thermal variations may induce breed-specific responses. In this context, NZW bucks appeared more sensitive, whereas LAP bucks showed a comparatively more stable pattern, more likely suggesting a higher homeostatic capacity in the local breed. This interpretation is consistent with previous studies in rabbits showing that reproductive responses to temperature may vary depending on genetic background, even under moderate thermal ranges [66].

These results highlight a significant interaction between environmental factors and genetic background in modulating reproductive performance, with photoperiod showing the most consistent associations with reproductive traits under the conditions of the present study.

While the present study provides valuable insights, a number of limitations should be taken into account. First, the study was conducted exclusively during the winter-to-spring period; therefore, the observed environmental responses cannot be directly extrapolated to summer conditions characterized by more pronounced heat stress. Second, fertility and prolificacy outcomes were not evaluated, preventing direct assessment of the practical reproductive consequences of the observed differences in semen characteristics. Third, microscopic sperm analyses were performed on pooled ejaculates, which precluded the evaluation of ejaculate order effects on sperm quality traits. Finally, testosterone concentrations were assessed at only two sampling points, providing limited insight into potential temporal or seasonal variations in endocrine activity.

## 5. Conclusions

In conclusion, the findings indicate that LAP bucks had a more favorable seminal profile than NZW bucks under the winter-to-spring conditions of this study, particularly regarding sperm concentration, viability and membrane integrity. Photoperiod was the environmental factor most consistently associated with reproductive variation during the study period, whereas THI exerted only limited, indirect effects on semen collection efficiency. The results also indicate breed-specific sensitivities to environmental cues; specifically, LAP bucks showed a more pronounced increase in sperm concentration with increasing photoperiod and more stable values under THI fluctuations than NZW bucks.

Overall, this study supports the potential integration of LAP bucks in rabbit AI programs under Algerian conditions and refines our understanding of how environment modulates male reproduction.

Further studies are nevertheless required to (i) elucidate the endocrine and molecular mechanisms driving these breed-specific responses, focusing on the roles of gonadotropins, melatonin, and oxidative status; (ii) monitor these patterns across a full annual cycle to confirm resilience under heat-stress conditions; and (iii) assess the impact on fertility outcomes and semen preservation efficiency under different storage conditions.

## Figures and Tables

**Table 1:** Monthly average meteorological data for the experimental period (mean ± SD).

Month	Temperature (°C)	Relative Humidity (%)	THI	Photoperiod (h)
January	12.73 ± 1.55	79.70 ± 8.08	12.80 ± 1.50	10.08 ± 0.19
February	12.72 ± 1.93	73.12 ± 10.06	12.85 ± 1.81	11.01 ± 0.27
March	16.11 ± 2.66	67.95 ± 9.78	15.90 ± 2.40	12.01 ± 0.30
April	19.05 ± 1.62	66.98 ± 10.32	18.61 ± 1.54	13.1 ± 0.29

THI: Temperature–Humidity Index.

**Table 2:** Semen collection traits and occurrence of qualitative characteristics of the ejaculate (%) according to the breed, ejaculate order, photoperiod and THI.

Variable		TNS	RS	UC	UR	BL	VL	G
Breed ^1^	LAP	412	95.90 ^a^	90.90 ^a^	3.50 ^b^	0.76 ^a^	4.8 ^a^	38.10 ^b^
	NZW	402	99 ^a^	92.70 ^a^	1.50 ^a^	1.01 ^a^	4.7 ^a^	12.40 ^a^
Ejaculate order ^1^	1st	407	99.02 ^b^	91.07 ^a^	3.23 ^b^	0.99 ^a^	4.7 ^a^	34.82 ^b^
	2nd	407	95.81 ^a^	92.54 ^a^	1.79 ^a^	0.77 ^a^	4.9 ^a^	15.21 ^a^
Photoperiod ^2^			1.74 (NS)	2.68 ***	0.47 ***	0.37 (NS)	0.96 (NS)	1.42 *
THI ^2^			0.80 (NS)	0.80 *	1.14 ***	1.20 (NS)	1.01 (NS)	0.94 (NS)

TNS: total number of solicitations, RS: response to solicitations, UC: useful collection, UR: contamination with urine, BL: contamination with blood, VL: volume ≤ 0.2 mL, G: gel plug. ^1^: Columns with different superscripts are significantly different (*p* < 0.05). ^2^: Odds Ratio. OR > 1 indicates increased odds of occurrence, OR < 1 indicates decreased odds of occurrence. NS: Odds Ratio not significantly different from 1; * *p* < 0.05; *** *p* < 0.001. None of the interactions involving Breed reached significance (*p* > 0.05).

**Table 3:** Libido of bucks and macroscopic traits of semen according to the breed, ejaculate order, photoperiod and THI.

Variable		T1 (s)	T2 (s)	T Total (s)	Total Volume (mL)	Gel-Free Volume (mL)	Gel Volume (mL)	pH
Breed (mean ± SD) ^1^	LAP	3.25 ± 4.03 ^a^	6.02 ± 5.83 ^a^	9.30 ± 9.28 ^a^	1.29 ± 0.86 ^b^	0.82 ± 0.37 ^a^	1.25 ± 1.49 ^b^	7.59 ± 0.26 ^a^
	NZW	4.59 ± 5.87 ^a^	6.88 ± 6.70 ^a^	11.43 ± 12.47 ^a^	0.96 ± 0.47 ^a^	0.89 ± 0.41 ^a^	0.58 ± 0.30 ^a^	7.66 ± 0.22 ^a^
Ejaculate (mean ± SD) ^1^	1st	4.49 ± 5.80 ^b^	7.05 ± 7.02 ^b^	11.55 ± 12.48 ^b^	1.24 ± 0.80 ^b^	0.84 ± 0.36 ^a^	1.10 ± 0.85 ^a^	7.63 ± 0.25 ^a^
	2nd	3.39 ± 4.10 ^a^	5.82 ± 5.37 ^a^	9.14 ± 9.16 ^a^	1.01 ± 0.56 ^a^	0.87 ± 0.42 ^a^	1.11 ± 2.13 ^a^	7.61 ± 0.23 ^a^
Photoperiod (β ± SE) ^2^		−1.23 ± 0.23 ***	−1.95 ± 0.34 ***	−3.19 ± 0.55 ***	0.07 ± 0.04 (NS)	0.03 ± 0.02 (NS)	−0.02 ± 0.15 (NS)	−0.13 ± 0.01 ***
THI (β ± SE) ^2^		−0.06 ± 0.09 (NS)	−0.14 ± 0.13 (NS)	−0.21 ± 0.22 (NS)	−0.01 ± 0.01 (NS)	−0.01 ± 0.008 (NS)	0.06 ± 0.06 (NS)	0.01 ± 0.005 *

T1: mounting latency, T2: ejaculation latency, T total: total latency. ^1^: Means within the same column with different superscripts are significantly different (*p* < 0.05). ^2^: Regression coefficient ± standard error. NS: Regression coefficient not significantly different from 0; * *p* < 0.05; *** *p* < 0.001. None of the interactions involving Breed reached significance (*p* > 0.05).

**Table 4:** Sperm quality and semen output of pooled ejaculates according to the breed, photoperiod and THI.

Variable	Breed			Photoperiod		THI	
	LAP (Mean ± SD)	NZW (Mean ± SD)	*p*	*β* ± SE ^1^	*p*	*β* ± SE ^1^	*p*
MM (0–9)	8.14 ± 1.00	7.92 ± 1.14	0.28	0.20 ± 0.08	0.02	0.06 ± 0.03	0.10
MI (0–4)	3.72 ± 0.49	3.72 ± 0.50	0.97	0.09 ± 0.04	0.03	0.01 ± 0.02	0.48
Mo tility (%)	81.64 ± 10.33	77.41 ± 12.38	0.07	3.75 ± 0.93	<0.001	−0.65 ± 0.38	0.09
Viability (%)	83.06 ± 9.95	78.57 ± 11.27	0.02	3.79 ± 0.85	<0.001	−0.28 ± 0.34	0.42
InMb (%)	86.25 ± 11.21	80.76 ± 13.95	0.04	6.02 ± 0.91	<0.001	−0.68 ± 0.37	0.07
Anomalies (%)	18.26 ± 7.28	19.62 ± 6.69	0.33	0.50 ± 0.63	0.43	−0.25 ± 0.25	0.33
Pool volume (mL)	1.49 ± 0.64	1.63 ± 0.72	0.24	0.17 ± 0.05	<0.001	−0.06 ± 0.02	0.01
Concentration (10^6^ spz/mL)	688.29 ± 292.51	505.24 ± 259.44	0.01	61.39 ± 20.83	<0.01	−9.23 ± 8.47	0.28
TSN (10^6^ spz)	1011.51 ± 628.72	788.73 ± 475.04	0.1	173.88 ± 43.21	<0.001	−39.82 ± 17.56	0.02
TMS (10^6^ spz)	843.45 ± 559.11	633.67 ± 417.33	0.08	163.67 ± 38.11	<0.001	−36.35 ± 15.49	0.02
AI doses	84.34 ± 55.91	63.37 ± 41.73	0.08	16.37 ± 3.81	<0.001	−3.63 ± 1.55	0.02

spz: spermatozoa, MM: mass motility, IM: individual motility, InMb: intact plasma membrane, TSN: total sperm number, TMS: total motile sperm number, AI doses: the number of artificial insemination doses/session. ^1^ Regression coefficient ± standard error of GLMM. Except for sperm concentration (see Table 5), all interactions involving breed were not significant (*p* > 0.05).

**Table 5:** Breed × environment interactions for sperm concentration.

Variable	Breed (NZW) × Photoperiod		Breed (NZW) × THI	
	***β*** **± SE ^1^**	***p***	***β*** **± SE ^1^**	***p***
Concentration (10^6^ spz/mL)	−42.73 ± 20.16	0.03	−17.48 ± 8.19	0.03

spz: spermatozoa. ^1^ Regression coefficient ± standard error of GLMM.

## Data Availability

The original contributions presented in this study are included in the article. Further inquiries can be directed to the corresponding author.
